# Scatter Radiation Distribution to Radiographers, Nearby Patients and Caretakers during Portable and Pediatric Radiography Examinations

**DOI:** 10.3390/bioengineering10070779

**Published:** 2023-06-29

**Authors:** Shing-Yau Tam, Yuen-Ying Fung, Sum-Yi Lau, Wang-Ngai Lam, Edward Ting-Hei Wong

**Affiliations:** Department of Health Technology and Informatics, The Hong Kong Polytechnic University, Kowloon, Hong Kong SAR 999077, China

**Keywords:** scattered radiation, radiation protection, portable radiography, pediatric radiography, medical radiation dose, X-ray examination, radiographer, caretaker

## Abstract

Scatter radiation from portable and pediatric X-rays could pose a risk to radiographers, nearby patients, and caretakers. We aim to evaluate the spatial scatter radiation distribution to the radiographers, nearby patients, and caretakers during common projections in portable and pediatric X-rays. We evaluated the three-dimensional scatter dose profiles of four and three commonly used portable and pediatric X-ray projections, respectively, by anthropomorphic phantoms and scatter probes. For portable X-ray, the AP abdomen had the highest scatter radiation dose recorded. Radiographer scatter radiation doses were 177 ± 8 nGy (longest cord extension) and 14 ± 0 nGy (hiding behind the portable X-ray machine). Nearby patient scatter radiation doses were 3323 ± 28 nGy (40 cm bed distance), 1785 ± 50 nGy (80 cm bed distance), and 580 ± 42 nGy (160 cm bed distance). The AP chest and abdomen had the highest scatter radiation dose in pediatric X-rays. Caretaker scatter radiation doses were 33 ± 1 nGy (50 cm height) and 659 ± 7 nGy (140 cm height). Although the estimated lens doses were all within safe levels, the use of shielding and caution on dose estimation by inverse square law is suggested to achieve the ALARA principle and dose optimization.

## 1. Introduction

Scatter radiation originates from the incident of X-ray after photons lose energy due to Compton interactions with objects. X-ray tube voltage (kVp) and the patient thickness have position correlations for the ratio between intensities of scatter and incident radiation [1]. Nevertheless, it is an important radiation exposure dose issue to radiological staff in various radiological modalities including general and portable X-rays [2]. This issue received more concerns among interventional radiologists and cardiologists as the scatter radiation dose may cause adverse cataract events [3]. Other than interventional radiology, scatter radiation may affect other parties in various settings. Scatter radiation from portable X-rays in wards could affect nearby patients and healthcare professionals [4,5]. While scatter radiation from pediatric X-rays may affect caregivers who help with the positioning of infants.

To reduce the scatter radiation exposure to radiological staff and other parties, increasing distance and shielding are the common strategies. It has been long assumed that the scatter radiation will decrease with increasing distance by inverse square law. However, the irregularity of scatter radiation may reduce the effects of radiation dose reduction by increasing distance as pointed out by a recent study [6]. For the use of shielding, it has been commonly found that physical shielding has not been used in ward settings due to weight and mobility concerns [2]. Therefore, scatter radiation in portable X-rays may affect nearby patients significantly due to proximity to the exposed patient and lack of physical shielding. The overcrowded wards in some hospitals such as Hong Kong public hospitals may cause the issue to be more prominent [7]. Recently, Chida [8] proposed the patient dose Optimization, Distance, Shielding, and Time (pdO-DST) as an updated policy for reducing occupational radiation dose of radiological medical workers, especially for interventional radiology workers. Also, the knowledge of occupational dose will help the staff to be more mindful of potential exposure risks and dose reduction methods. Similar knowledge in portable and pediatric radiography should be strengthened to achieve better occupational radiation dose reductions.

In recent years, the use of portable X-rays has significantly increased by 1.7-fold due to COVID-19 [9]. This study, with other studies, commonly used thermoluminescent dosimeters (TLD) to estimate occupational doses [4,5,9]. These studies concluded that the average reported doses remained significantly lower than the annual occupational whole-body dose limit or lens dose limit of 20 mSv set by the International Commission on Radiological Protection (ICRP) [10]. A Japanese multicenter study conducted by Matsubara et al. [11] estimated the eye lens dose (H_p_(3)) of physicians and other medical staff engaged in interventional radiology procedures. They found that the average annual H_p_(3) value of physicians was 25.5 ± 38.3 mSv and 9.3 ± 16.6 mSv for left and right eyes, respectively. While the corresponding values for other medical staff were 3.7 ± 3.1 mSv and 3.2 ± 2.7 mSv for left and right eyes, respectively. The results signify the possibility of exceeding of eye lens dose limit among radiation workers. While another Japanese group conducted multicenter research on the occupational dose of lenses for the staff involved in endoscopic retrograde cholangiopancreatography (ERCP) [3]. There was a total of 631 ERCPs measured in the research, small-sized TLDs were inserted inside and outside of the lead glasses to estimate the annual occupational lens dose and the shielding rates of the lead glasses. The study concluded that the median estimated annual occupational lens dose and the shielding rates to be 2.2–3.7 mSv and 44.6–66.3%, respectively for different staff roles. As TLD usually measures a fixed position on the body when conducting X-ray examinations for longer monitoring periods with a large lower detection limit, spatial measurement of scatter radiation at a three-dimensional level should be conducted by a scatter radiation detector that provides real-time values due to the irregularity of scatter radiation. Moreover, lens dose is a common concern raised by different studies [3,12,13] and warrants deeper studies on the lens dose of various radiological procedures with a risk of scatter radiation exposure including portable and pediatric X-rays. Furthermore, according to the as low as reasonably achievable (ALARA) principle under the optimization criteria in ICRP and International Radiation Protection Association (IRPA) recommendations, the radiation dose at a very low level may still induce stochastic effect, leading to DNA damage and cancer [14]. More information on spatial scatter radiation could improve the planning to achieve ALARA. Also, the information may be beneficial for the learning of appropriate radiation protection methods among radiological technology students [15].

The Monte-Carlo simulation has been suggested to estimate the spatial scatter radiation distribution in different radiological procedures including portable X-ray [16] and interventional radiology [17]. However, Monte-Carlo simulation cannot fully replace the use of phantom and scatter probes complying with the International Electrotechnical Commission (IEC) 60,601 and Title 21 of the Code of Federal Regulations (CFR) for measuring scatter radiation [18]. Also, there is no study to date for studying the scatter radiation to the caretaker (usually the infant’s parent) during the aid of infant radiological examinations [19]. During infant radiological examinations, the parent is often asked to aid the positioning in the examination room, and a lead apron is usually provided, but lens protection is usually absent. Therefore, our research aims to evaluate the spatial scatter radiation distribution to the radiographers, nearby patients, and caretakers during common projections in portable and pediatric X-rays.

## 2. Materials and Methods

### 2.1. Experimental Materials and Machines

The experiments were conducted in the radiography clinic at the Hong Kong Polytechnic University. For portable X-ray, whole body anthropomorphic phantom of adult size (PBU-60, Kyoto Kagaku Co., Ltd., Kyoto, Japan) and Mobilett XP (Siemens Healthineers, Eriangen, Germany) were employed. For pediatric X-ray, newborn infant size (PBU-80, Kyoto Kagaku Co., Ltd.) and DRX-Evolution Plus (Carestream Health, Rochester, NY, USA) were employed. The spatial scatter radiation RTI Scatter Probe (RTI Group, Mölndal, Sweden, photon energy range of use: 10–150 keV, accuracy: ±10% or ±0.3 μGy/h (ISO N20-N150)) was employed with 100 cm^2^ detector setting. The use of a 100 cm^2^ detector conforms to IEC 60,601 and Title 21 of CFR. The calibration of the meter was conducted by the manufacturer. The scatter probe was in the validity period of calibration according to manufacturer data. If the recorded value exceeded the detection limit, the mAs were reduced and the scatter radiation doses were calculated proportionally. Loaded computed radiography (CR) cassette (Fujifilm Corporation, Tokyo, Japan) was placed according to the local positioning practice.

### 2.2. Exposure Settings and Measurement Points for Portable X-ray

**Figure 1:**
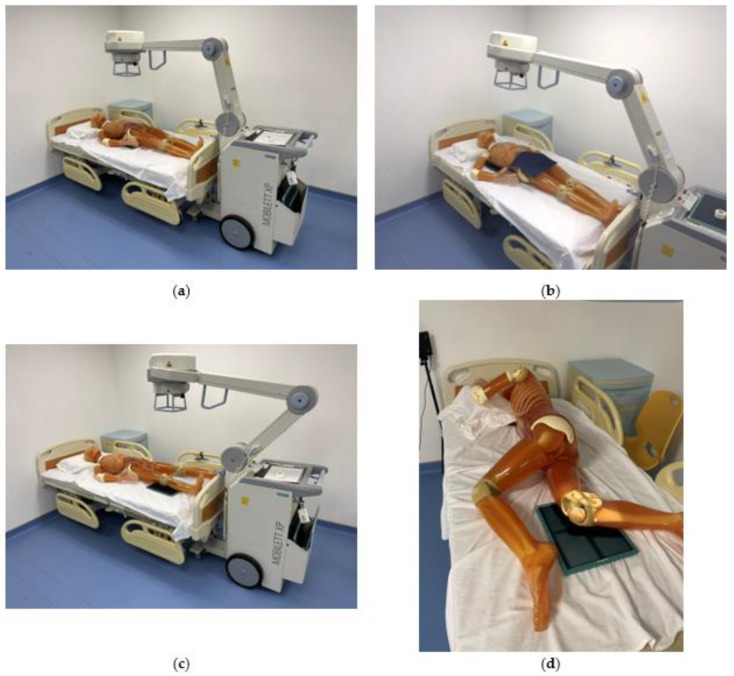
Positioning of phantom for portable X-ray scatter radiation evaluation. (**a**) AP abdomen; (**b**) AP chest; (**c**) AP right knee; (**d**) lateral right knee.

We selected 4 commonly used portable X-ray projections, including anteroposterior (AP) abdomen (Figure 1a), AP chest (Figure 1b), AP, and lateral right knee (Figure 1c,d) for evaluation. The exposure settings including field size, tube voltage (kVp), tube current-time product (mAs), and source-to-image-receptor distance (SID) were listed in Table 1. The bed height was 75 cm. Suitable collimations were applied according to the local practice.

**Figure 2:**
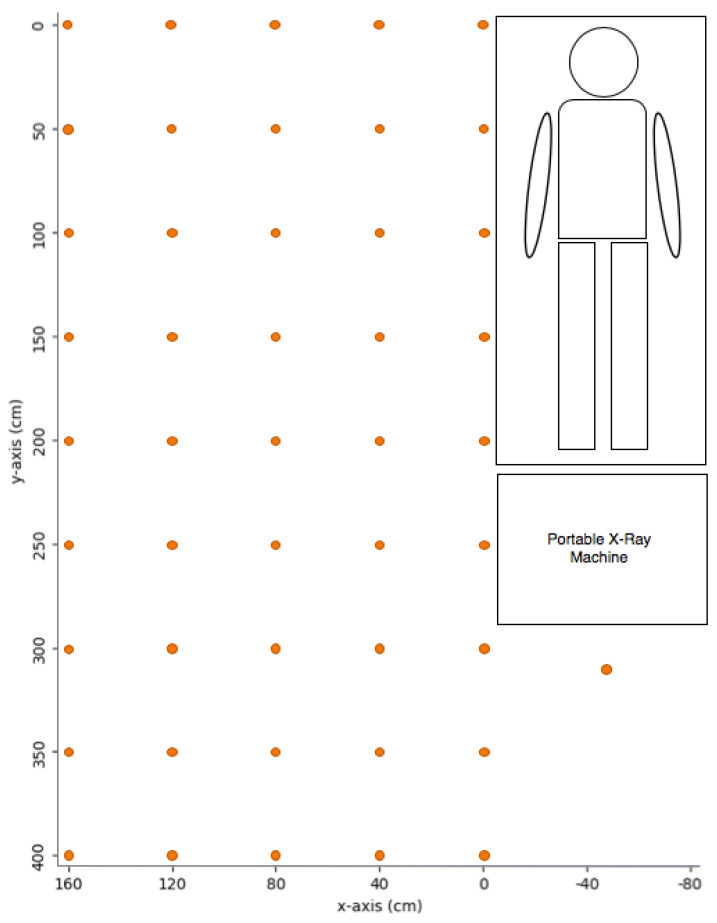
Measurement points of scatter radiation in portable X-ray projections.

For the measurement points, a three-dimensional coordinate plot has been set in the portable X-ray room (Figure 2). The x, y, and z coordinates represent the horizontal distance from the bedside, the distance from the bed top, and the height, respectively. Limited by the room construction and the cord length of the portable X-ray machine, 40 cm intervals up to 160 cm and 50 cm intervals up to 400 cm were measured for x and y, respectively. The upper limit y was set according to the longest cord extension of the portable X-ray machine. The dose behind the portable machine, i.e., 100 cm from the bed end and in the midline of the bed (x = −50 cm, y = 300 cm) was also measured. For height, 50, 100, 150, and 200 cm were selected for measurement. There was a total of 184 measurement points for each portable X-ray projection. The scatter probe was orientated toward the X-ray tube at all measurement points.

### 2.3. Exposure Settings and Measurement Points for Pediatric X-ray

**Figure 3:**
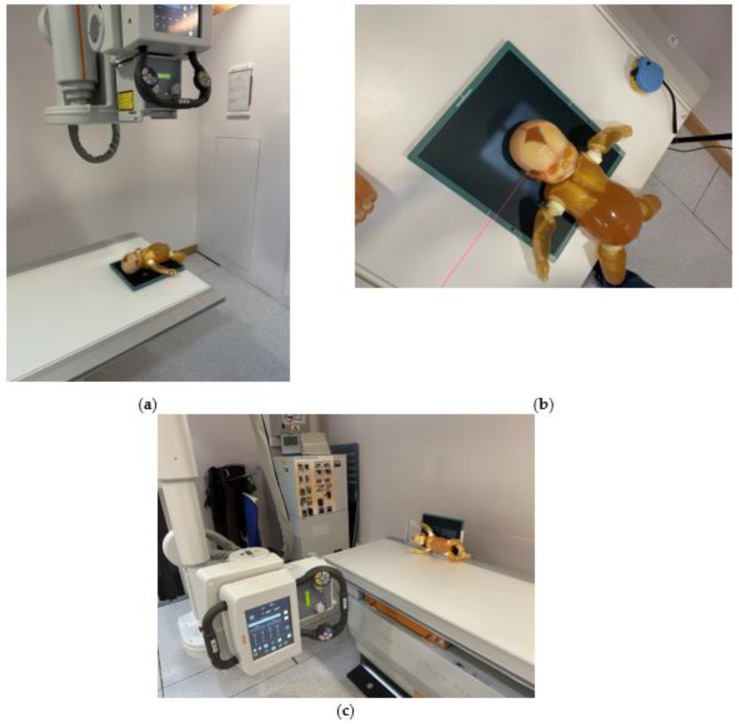
Positioning of phantom for pediatric X-ray scatter radiation evaluation. (**a**) AP chest and abdomen; (**b**) AP skull; (**c**) abdomen in right lateral decubitus view.

We selected three commonly used pediatric X-ray projections for evaluation, including AP chest and abdomen (Figure 3a), AP skull (Figure 3b), and abdomen in right lateral decubitus view (Figure 3c). The exposure settings including field size, kVp, mAs, and SID were listed in Table 2. The table height was 70 cm. Suitable collimations were applied according to the local practice.

**Figure 4:**
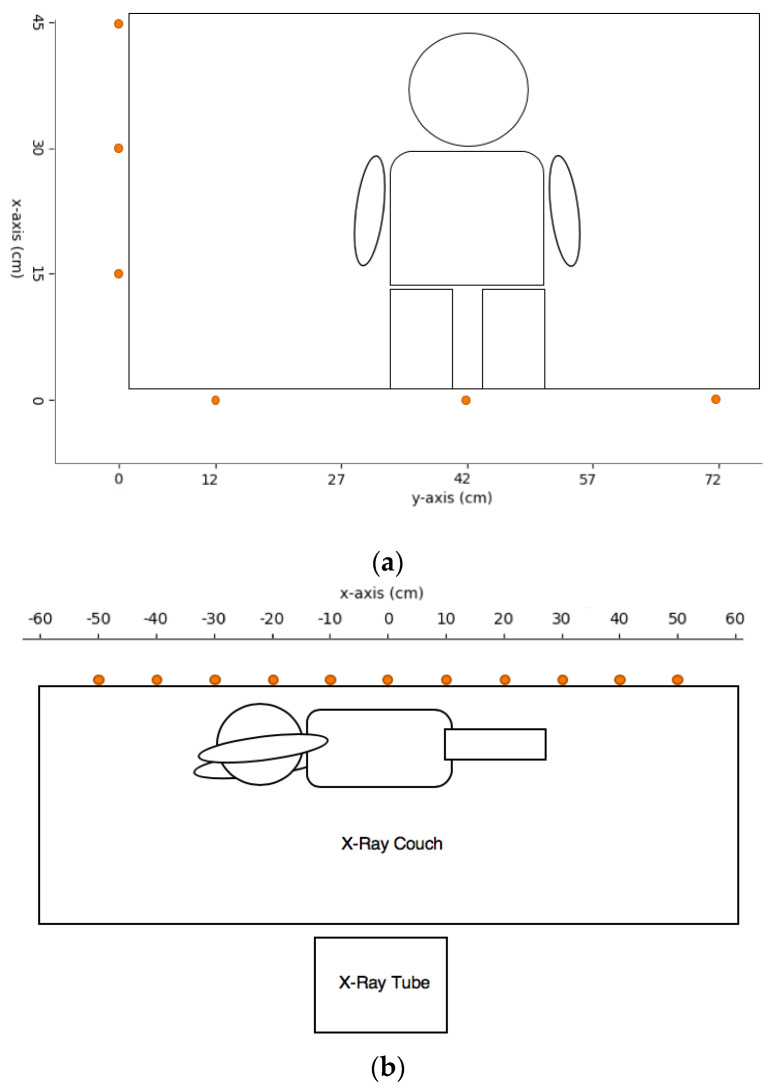
Measurement maps for (**a**) AP chest and abdomen and AP skull; (**b**) Abdomen in lateral decubitus view of pediatric X-ray.

For AP chest and abdomen, and AP skull, the measurement points were set adjacent to the table at 15, 30, and 45 cm for the left table side, and midline, +30 and −30 cm from the phantom for the table-end side (Figure 4a). For the abdomen in the right lateral decubitus view, the measurement points were set at 10 cm intervals from the midline of the cassette to 50 cm toward the caudal (denoted as positive) and cranial (denoted as negative) sides (Figure 4b). The measurement heights were 50, 100, 140, 150, 160, and 170 cm to correspond to the eye lens dose.

### 2.4. Data Represenation and Statistical Analysis

Each measurement point was repeated at least three times. Key measurement points were presented as mean ± standard deviation. The spatial scatter dose distribution is presented by Python.

## 3. Results

### 3.1. Portable X-ray

**Figure 5:**
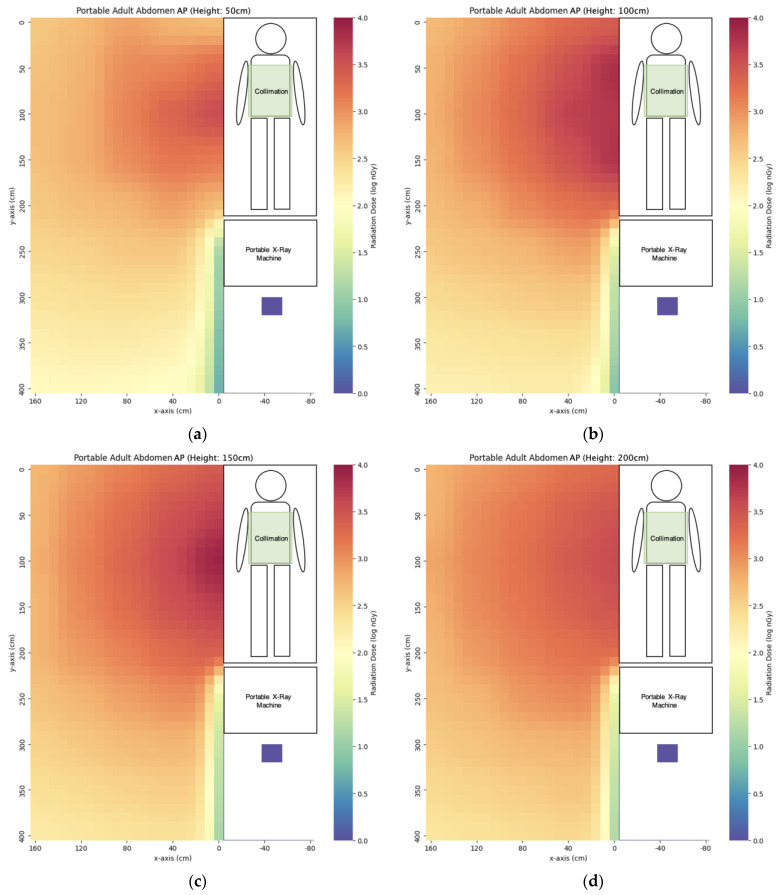
The scatter dose distribution of portable AP abdomen at (**a**) z = 50 cm; (**b**) z = 100 cm; (**c**) z = 150 cm; (**d**) z = 200 cm. Data represented by mean, N ≥ 4.

**Figure 6:**
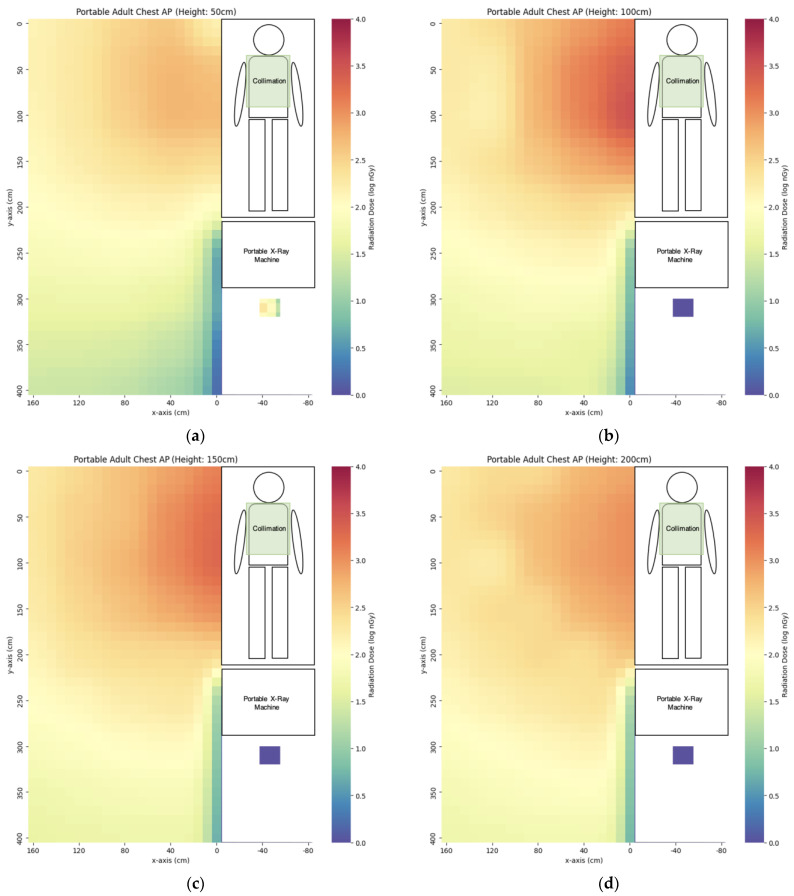
The scatter dose distribution of portable AP chest at (**a**) z = 50 cm; (**b**) z = 100 cm; (**c**) z = 150 cm; (**d**) z = 200 cm. Data represented by mean, N ≥ 4.

**Figure 7:**
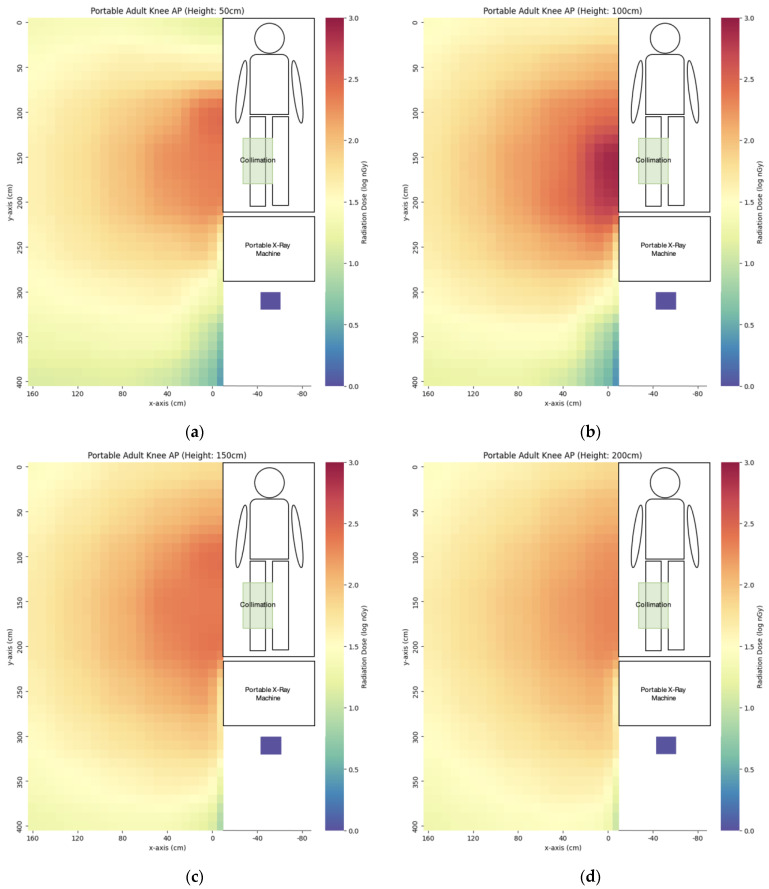
The scatter dose distribution of portable AP right knee at (**a**) z = 50 cm; (**b**) z = 100 cm; (**c**) z = 150 cm; (**d**) z = 200 cm. Data represented by mean, N ≥ 4.

**Figure 8:**
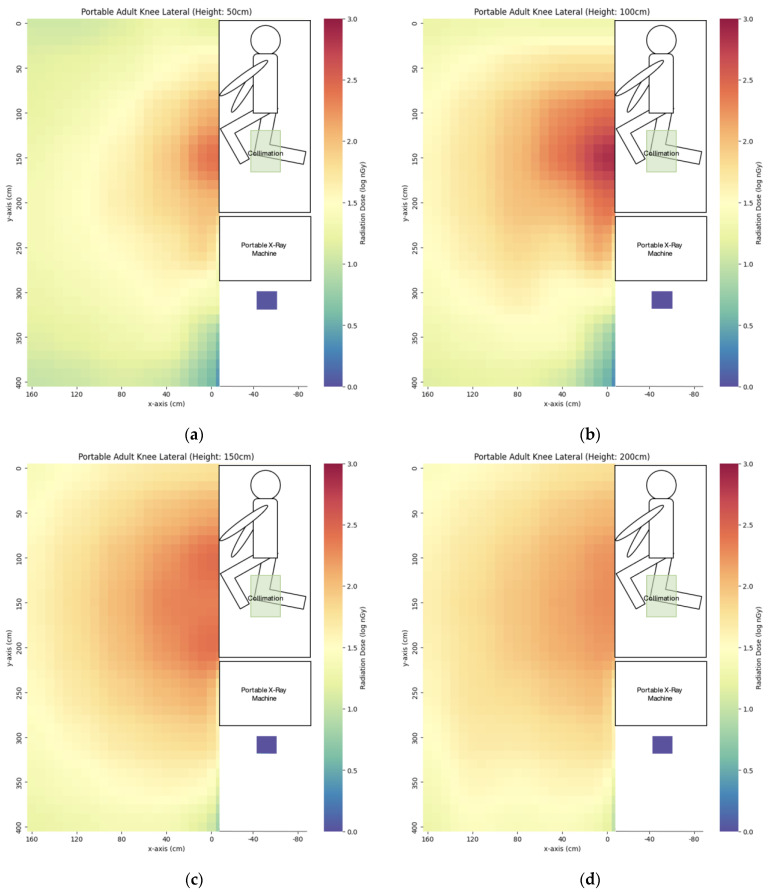
The scatter dose distribution of portable lateral right knee at (**a**) z = 50 cm; (**b**) z = 100 cm; (**c**) z = 150 cm; (**d**) z = 200 cm. Data represented by mean, N ≥ 4.

We have evaluated the spatial scatter radiation dose distribution of four commonly used portable X-ray projections. The scatter dose distribution maps are listed in Figure 5, Figure 6, Figure 7 and Figure 8. In general, the abdomen X-ray has the highest scatter dose while the lateral knee X-ray has the lowest scatter dose. For the key measurement points related to the radiographer’s dose, the points representing the cord extended to the longest length with eye level height (x = 160 cm, y = 400 cm, z = 150 cm) and hiding behind the portable X-ray machine (x = −50 cm, y = 310 cm, z = 50 cm) were chosen for comparison (Table 3). Except for the lateral right knee, the doses for hiding behind the portable X-ray machine were lower than that of the longest cord extension in the other three projections. The percentage of the dose for hiding behind the portable X-ray machine ranged from 7–141% of that of the longest cord extension. For the key measurement points for nearby patient dose (Table 4), we chose y = 50 cm and 100 cm height (z) to evaluate eye lens dose with the assumption of the lying position of the nearby patient. The dose at detector distance from the irradiated phantom bedside (x) of 40 cm was the highest in the abdomen (3323 ± 28 nGy) while the dose for the lateral right knee was the lowest (67 ± 1 nGy). After increasing x to 80 cm, the dose dropped to 46–75% of the dose value at x = 40 cm. When the x distance was extended to 160 cm, the dose dropped to 31–56% of the dose value at x = 80 cm.

### 3.2. Pediatric X-ray

**Figure 9:**
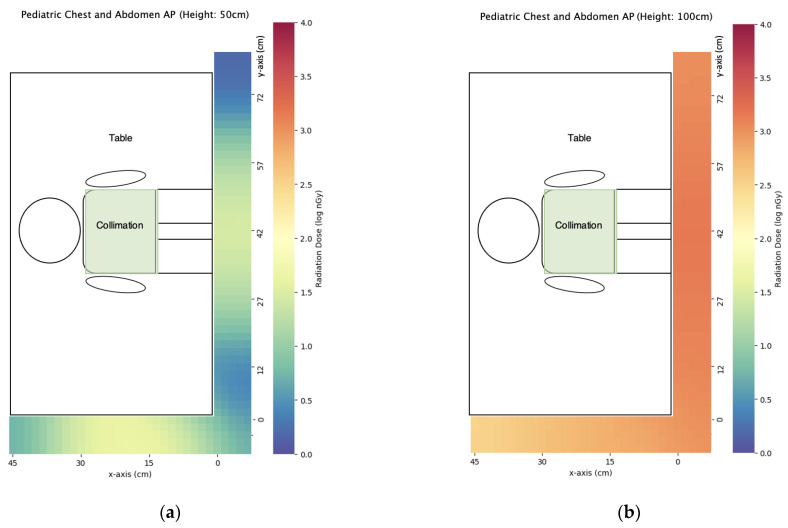

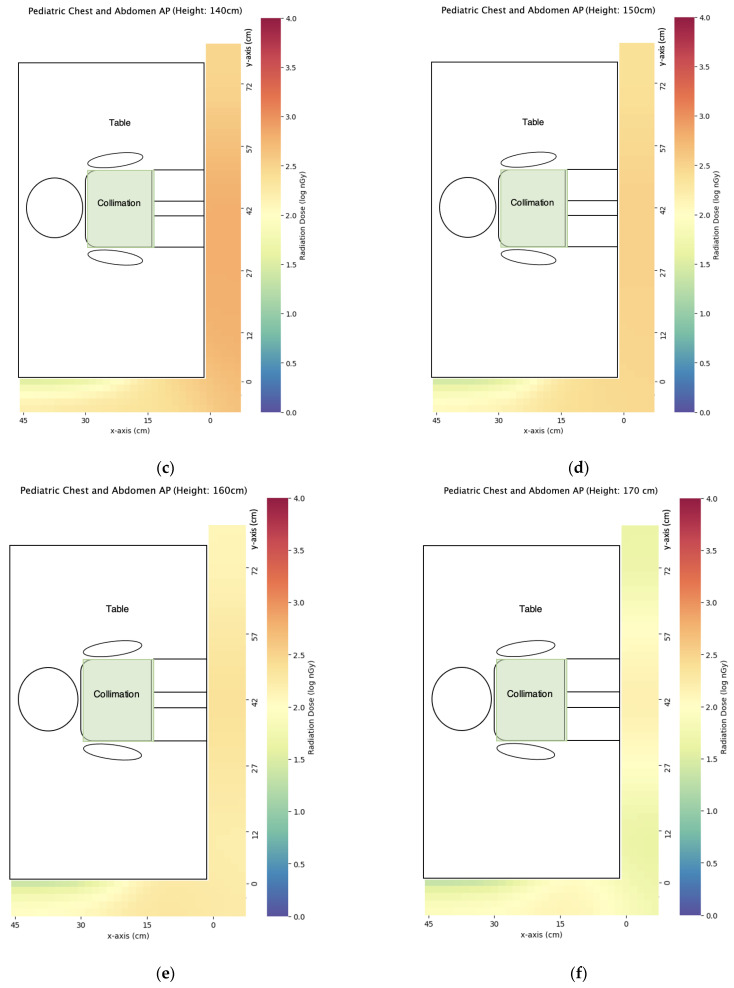
The scatter dose distribution of pediatric AP chest and abdomen at (**a**) z = 50 cm; (**b**) z = 100 cm; (**c**) z = 140 cm; (**d**) z = 150 cm; (**e**) z = 160 cm; (**f**) z = 170 cm. Data represented by mean, N ≥ 4.

**Figure 10:**
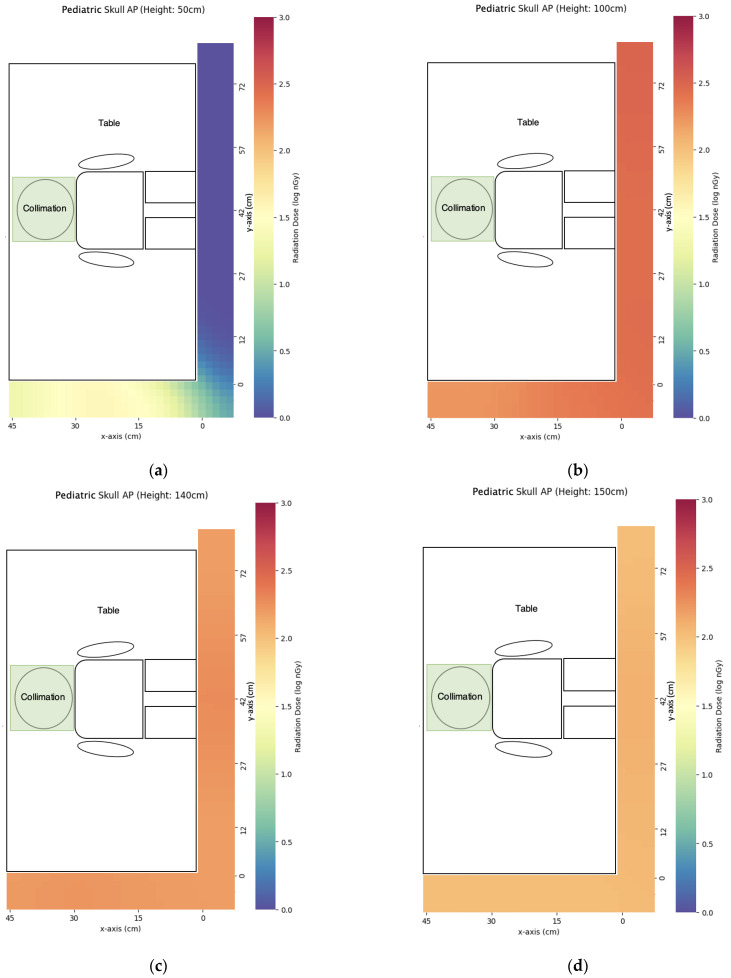

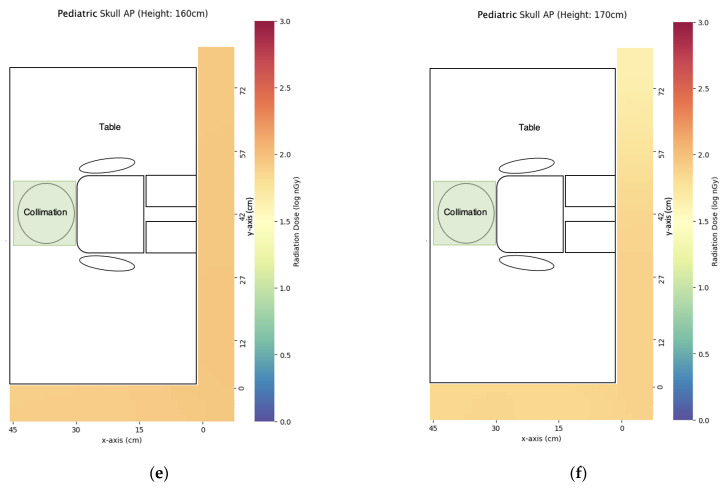
The scatter dose distribution of pediatric AP skull at (**a**) z = 50 cm; (**b**) z = 100 cm; (**c**) z = 140 cm; (**d**) z = 150 cm; (**e**) z = 160 cm; (**f**) z = 170 cm. Data represented by mean, N ≥ 4.

**Figure 11:**
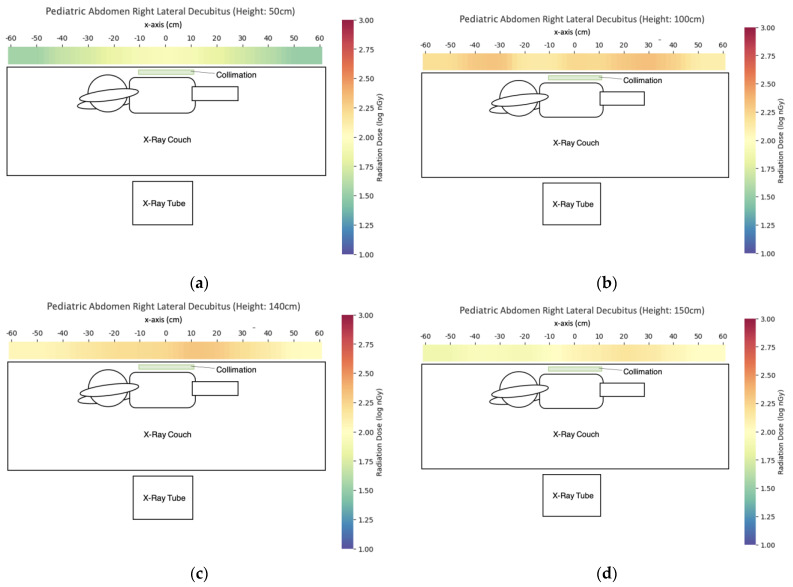

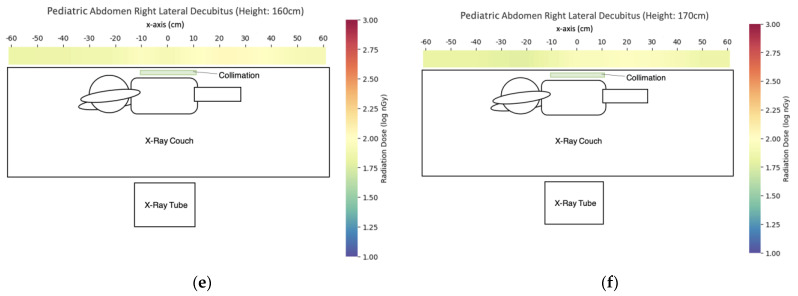
The scatter dose distribution of pediatric abdomen in right lateral decubitus view at (**a**) z = 50 cm; (**b**) z = 100 cm; (**c**) z = 140 cm; (**d**) z = 150 cm; (**e**) z = 160 cm; (**f**) z = 170 cm. Data represented by mean, N ≥ 4.

We have evaluated three commonly used pediatric X-ray projections with scatter dose distribution maps listed in Figure 9, Figure 10 and Figure 11. In general, the scatter dose for AP chest and abdomen was the highest while the scatter dose for the other two projections is similar. Using the central points (x = 0 cm, y = 42 cm for AP skull and AP chest and abdomen, y = 0 cm for abdomen in lateral decubitus view) for comparison of caretaker dose (Table 5), the scatter dose for hiding lower than the table height (z = 50 cm) and greater height (z = 170 cm) had the lower doses compared with those just above than the table height (z = 100 cm). Except for the abdomen in the right lateral decubitus view, z = 50 cm had a lower scatter dose than z = 170 cm. At the eye level of z = 150 cm, AP chest and abdomen had the highest scatter dose of 338 ± 7 nGy while the scatter doses of AP skull and abdomen in right lateral decubitus view were 127 ± 2 nGy, and 117 ± 3 nGy, respectively. The trends were similar for z = 140–170 cm.

## 4. Discussion

Scatter radiation is the major source of occupational dose for radiographers [18]. It also poses a risk to nearby patients in the ward during portable X-ray examinations and caretakers during pediatric X-ray examinations. Although a previous study [16] evaluated the scatter radiation in portable chest examination by the Monte-Carlo simulation, they were targeting the radiation profiles near the phantom but rather the impact of scatter dose on nearby patients and radiographers. In this study, we aim at evaluating the spatial scatter radiation distributions of a range of commonly used projections in portable and pediatric X-ray examinations for understanding the doses to radiographers, nearby patients, and caretakers. Although radiographers and caretakers are often provided lead aprons for protection, other radiosensitive organs that are not protected by lead aprons such as the lens may be at risk for excessive radiation exposure. In a recent African study [20], there was an inadequate understanding of radiation protection measures including the use of lead aprons, and work experience was found to be a significant factor for lower occupational dose. This signifies more studies and education programs are required in the field of scatter radiation.

We have several notable findings from the results. First, hiding behind the portable X-ray machine could achieve better scatter dose reduction when compared with the longest cord extension in the majority of the evaluated projections. The reduction by hiding behind the portable X-ray machine was particularly more in the AP abdomen and AP chest while the benefits were limited for knee projections (Table 3). This demonstrated the use of shielding is more useful than long-distance unless the position is in proximity to the X-ray field. If the cord could be extended beyond a wall and allow the radiographer to control the exposure around the corner, the dose to the radiographer may be further decreased but this requires further investigations to confirm. However, we found that the decrease achieved by hiding behind the portable X-ray machine was already very good as only 3–24 nGy were recorded in our studied projections. In addition to hiding behind the portable X-ray machine, radiation-absorbing pads made from a composite material of lead, barium, and tungsten in lead equivalence of 0.5 mm may be considered. Koenig et al. [21] applied the said radiation-absorbing pads attached to the X-ray tube of the fluoroscopy machine. Results showed that a radiation-absorbing pad could achieve significant scatter radiation dose reduction of up to 80.6% and is especially beneficial for the upper body parts. Similar techniques may be considered in portable X-ray machines to achieve further scatter dose reduction. Also, personal radiation protective equipment such as lead glasses and non-lead protective aprons may be considered for radiographers with heavy portable X-ray duties. In interventional cardiology procedures, wearing lead glasses of 0.07 mm lead equivalent could achieve a 60% shielding effect for the lens [22]. However, there is no related study done on the shielding effect of lead glasses for portable X-ray workers, and thus further studies are needed to confirm the benefits of wearing lead glasses. While for non-lead protective aprons, a recent short report by Kato et al. [23] has found similar shielding effects of non-lead protective aprons compared to lead protective aprons with the same lead equivalent thickness but offered higher comfortably for physicians in interventional radiology procedures. This method could also be applied to radiographers with heavy portable X-ray duties for increasing their acceptance of wearing protective aprons.

The second finding is that the estimated eye lens dose for the nearby patients generally follows the inverse square law. After adding the 50 cm for half of the bed width and considering the center of the X-ray field, the dose decrease from x = 40 cm to x = 80 cm in AP abdomen and AP chest were very close to the theoretical dose decrease to 47.93% (AP abdomen: 54%, AP chest: 46%) (Table 4). While for the dose decrease from x = 80 cm to x = 120 cm, the degree of dose decrease was more variated compared to the theoretical dose decrease to 38.32% (AP abdomen: 56%, AP chest: 26%). As the larger field size to distance ratio and the presence of other objects such as bed and wall could affect the reliability of scatter radiation prediction by inverse square law, the estimated decrease of radiation dose by inverse square law should be applied more conservatively to minimize the effect of scatter radiation on other persons nearby for achieving the ALARA principle. Nevertheless, it is worth noting that the lens doses of nearby patients in portable X-ray examination were up to the level of several µSv in our investigated projections as recorded in AP abdomen due to larger field size and greater body thickness, which are still far less than the lens dose limit of 20 mSv set by ICRP [10].

The third finding is that pediatric X-rays could achieve a similar scatter dose to the caretakers as portable X-rays to the nearby patients, with the maximum dose recorded in pediatric AP chest and abdomen X-rays. The reasons for pediatric AP chest and abdomen X-rays to produce higher scatter doses include closer to the detector position from the center of the X-ray field compared with AP skull, and the presence of an X-ray film cassette as a radiation attenuation object between the detector and incident X-ray field for abdomen in right lateral decubitus view. The height of eye level could have a great impact on scatter dose (Table 5). Taking pediatric AP chest and abdomen as an example, the dose at z = 150 cm was 51.21% of that at z = 140 cm while the dose at z = 160 cm was 73.33% of that at z = 150 cm. Although the predicted lens dose to caretakers was still low at the level of several hundred nSv, the caretakers could wear lens shields especially when their views are inevitably directed on the pediatric patients during the exposure or keeping under the X-ray table during exposure to reduce lens dose for achieving the ALARA principle and dose optimization.

For the limitation of this study, although we have tried to mimic the ward setting of a portable X-ray room, we conducted the experiments in a room that is smaller than usual wards. Therefore, the value may not be directly translated to the actual lens dose of the nearby patients as the bed settings differ among different hospitals and wards. However, our results were still useful for deriving the concepts in the spatial scatter radiation profiles of some commonly used portable X-ray projections. Moreover, for the smaller dose values detected at a level of several nGy, the accuracy may be lower as the scattering probe has an accuracy of 0.3 µGy/h and a sampling rate of 1–300 Hz. While the scattering probe was in the validity period of calibration, we repeated the same measurement points at least three times and found the standard deviations were all within reasonable ranges. Thus, the results were still considered with adequate integrity.

## 5. Conclusions

Our study has evaluated the spatial scatter radiation profiles of several commonly used portable and pediatric X-ray projections for understanding the scatter dose to radiographers, nearby patients, and caretakers. Although the values recorded are still considered safe for lens dose, hiding behind a portable X-ray machine for radiographers, caution on the dose estimation by inverse square law on nearby patient dose, and wearing lens shield or hiding under X-ray table for caretakers are suggested to achieve the ALARA principle and dose optimization.

## Figures and Tables

**Table 1:** Exposure factors for the evaluated portable X-ray projections.

Projection	Field Size	kVp	mAs	SID
AP abdomen	35 cm × 43 cm	81	11	100 cm
AP chest	35 cm × 43 cm	81	11	100 cm
AP right knee	24 cm × 30 cm	60	5	100 cm
Lateral right knee	24 cm × 30 cm	60	5	100 cm

**Table 2:** Exposure factors for the evaluated pediatric X-ray projections.

Projection	Field Size	kVp	mAs	SID
AP chest and abdomen	14 cm × 25 cm	65	3.2	110 cm
AP skull	14 cm × 14 cm	65	3.2	110 cm
Abdomen in lateral decubitus view	14 cm × 20 cm	65	3.2	110 cm

**Table 3:** Dose of the key measurement points for radiographer dose in the evaluated portable X-ray projections (nGy).

Projection	Longest Cord Extension	Hiding behind the Portable X-ray Machine
AP abdomen	177 ± 8	14 ± 0
AP chest	43 ± 1	3 ± 0
AP right knee	19 ± 1	5 ± 0
Lateral right knee	17 ± 1	24 ± 0

**Table 4:** Dose of the key measurement points for nearby patient dose in the evaluated portable X-ray projections (nGy).

Projection	x = 0 cm	x = 40 cm	x = 80 cm	x = 120 cm	x = 160 cm
AP abdomen	6760 ± 251	3323 ± 28	1788 ± 50	1008 ± 36	580 ± 42
AP chest	2588 ± 422	1302 ± 4	604 ± 14	160 ± 10	187 ± 6
AP right knee	117 ± 2	89 ± 1	66 ± 3	52 ± 1	37 ± 0
Lateral right knee	69 ± 1	67 ± 1	50 ± 1	34 ± 0	25 ± 1

**Table 5:** Dose of the key measurement points for caretaker dose in the evaluated portable X-ray projections (nGy).

Projection	z = 50 cm	x = 100 cm	x = 140 cm	x = 150 cm	x = 160 cm	x = 170 cm
AP chest and abdomen	33 ± 1	1490 ± 109	659 ± 7	338 ± 7	248 ± 1	170 ± 7
AP skull	1 ± 0	532 ± 14	194 ± 2	127 ± 2	90 ± 1	73 ± 1
Abdomen in right lateral decubitus view	86 ± 1	172 ± 11	167 ± 4	117 ± 3	79 ± 2	87 ± 4

## Data Availability

Not applicable.
